# High risk sexual behaviours associated with traditional beliefs about gender roles among men interested in medical male circumcision in South Africa

**DOI:** 10.1186/s12981-021-00359-7

**Published:** 2021-06-22

**Authors:** Candice M. Chetty-Makkan, Jonathan M. Grund, Evans Muchiri, Matt A. Price, Mary H. Latka, Salome Charalambous

**Affiliations:** 1 The Aurum Institute, Aurum House, The Ridge, 29 Queens Road, Parktown, Johannesburg, South Africa; 2 Division of Global HIV & TB, Centers for Disease Control and Prevention, Pretoria, South Africa; 3 School of Public Health, Faculty of Health Sciences, University of the Witwatersrand, Johannesburg, South Africa; 4 IAVI, New York, USA; 5 Department of Epidemiology and Biostatistics, University of California San Francisco, San Francisco, CA USA; 6 Health Economics and Epidemiology Research Office (HE2RO), Johannesburg, South Africa

**Keywords:** HIV risk, Men, Beliefs about gender roles, South Africa, Circumcision

## Abstract

**Background:**

Beliefs about gender roles and high-risk sexual behaviours underlie the human immunodeficiency virus (HIV) epidemic in South Africa. Yet, there is limited information on the relationships between beliefs about gender roles and risky sexual behaviours. Few studies have explored the association between beliefs about gender roles, high risk sexual behaviour, and health-seeking behaviour among men.

**Methods:**

We investigated associations between gender beliefs (dichotomised as traditional or progressive) and high-risk sexual behaviour among South African men presenting for medical male circumcision (Apr 2014 to Nov 2015).

**Results:**

Of 2792 enrolled men, 47.4% reported traditional gender beliefs. Participant ages ranged between 18–46 years (median age 26 years; interquartile range, 21–31 years). Most participants had at least one sex partner over the last 12 months (68.2%). Younger men (18–24 years old vs. 25–46 years old) (odds ratio [OR], 1.5 [95% confidence interval (CI) 1.0–2.0]), those with multiple partners ([OR], 1.5 (CI) 1.3–1.8]) and participants unsure of their last partner’s HIV status (OR, 1.4 [95% CI 1.1–1.7]) were more likely to have traditional beliefs on gender roles.

**Conclusion:**

Young men with traditional beliefs on gender roles may be more likely to engage in high-risk sexual behaviour and could be good candidates for HIV prevention programmes. N = 206 (max 350)

*Trial registration* Name of registry: Clinicaltrials.gov; Trial registration number: NCT02352961; Date of registration: 30 January 2015 “Retrospectively registered”; URL of trial registry record: https://www.clinicaltrials.gov/

## Introduction

Beliefs about gender roles and high risk sexual behaviours may underlie the human immunodeficiency virus (HIV) epidemic in South Africa [1–7]. Beliefs about gender roles are dynamic, context-specific [7–11], socially constructed [7, 12] and influence how men interact with their sexual partners. Traditional roles of men are characterized by the beliefs that men are the primary bread-winners [13] and superior to women [13, 14]. Progressive beliefs about gender roles are in support of gender equality [13] and promote participation in HIV prevention for individuals and families [15]. Yet there is limited information on the relationships between beliefs about gender roles and risky sexual behaviours.

According to the 2017 South African national survey, black South African men aged 45–49 years had higher HIV risk [16] than women in the same age group. Men also had lower HIV treatment coverage (41%) than women (52%) and accounted for 58% of AIDS-related deaths [2]. Sexual behaviours that increase HIV risk among men include independent or impulsive sexual decision-making [6, 7, 10, 11, 17, 18], refusal to test for HIV [19, 20], not knowing or denial of partner HIV status [21], inconsistent condom use [22, 23], and having multiple sexual partners [15, 22–27]. Yet few studies have explored the association between beliefs about gender roles, high risk sexual behaviour, and health-seeking behaviour among men.

Medical male circumcision (MMC) reduces the risk of heterosexual transmission of HIV [28]. However, uptake of HIV prevention interventions, including MMC, remains low [29, 30]. The UNAIDS 2017 report showed that 37% of adult men in South Africa are living with HIV [31], and the South African national survey indicated that only 31.8% men aged 15–64 years were circumcised [32]. These statistics highlight the need to identify men at high risk for HIV and involve them in HIV prevention activities, such as MMC.

To help identify men at high risk of HIV, we used a scale on gender roles to determine whether traditional beliefs are associated with high risk sexual behaviour among South African men.

## Methods

### Description of the study area

The parent study that collected the data for this analysis was conducted at a health clinic in Ekurhuleni North, a peri-urban area in Gauteng Province, South Africa. The clinic provides services to adolescent and adult men. Routine sexual health services include HIV counselling and testing, provision of condoms, screening for sexually transmitted infections (STIs), linkage to treatment for HIV or STIs, and MMC.

### Parent study

Retrospective data for this analysis were derived from parent study *Imbizo* (which is the IsiZulu word for traditional forums where elders discuss important issues with the community. IsiZulu is a language spoken in South Africa) [33], which developed and evaluated an intervention to increase demand for circumcision among HIV-uninfected men aged 25–49 years. During enrolment of the Imbizo study, quantitative behavioural risk data during were collected from participants. In phase one (April 1–September 30, 2014), we identified characteristics of men presenting for MMC. In phase two (June 22–November 30, 2015) we implemented an intervention strategy and determined whether this intervention increased the number of men (aged ≥ 25 years) presenting for MMC at the clinic. The intervention included infrastructure changes that physically separated adult from adolescent males inside the facility, an exclusive health club, adult-specific community awareness materials, and community discussions [33]. Details and outcomes of the intervention are described elsewhere [33]. For this report, we analysed data from all men aged 18–49 years who enrolled during both phases.

### Enrolment procedures

For this analysis, we used cross-sectional data from the parent study first visit. We analysed data from the structured behavioural risk questionnaires that were administered during enrolment. Eligibility criteria for our analysis included age 18–49 years at enrolment, enrolment in the parent study, eligible for circumcision at screening, and ability to communicate in English, IsiZulu, or Sepedi. Following informed consent, trained interviewers administered the structured behavioural risk questionnaire to all participants before the circumcision procedure.

### Data collection tools

The beliefs about gender roles Likert scale (Table 1) is an eight-item index with four response options, derived and previously validated from research conducted in South Africa, where the estimate of the internal consistency (Cronbach alpha) of the scale was good [34]. In addition we administered a structured behavioural risk questionnaire that included demographic information (e.g., age, employment, and alcohol or drug use), number of partners in the last 12 months, age at sexual debut, sexual partner history, and condom use at last sex act.

**Table 1:** Beliefs about gender roles were measured using an eight-item index with four-level response options

No		Progressive		Traditional	
		Strongly Disagree	Disagree	Agree	Strongly agree
1	Men have many lovers because it is in their nature to do so	1	2	3	4
2	Men have lovers to get energy to satisfy their primary partners	1	2	3	4
3	Women these days say that they need to have more than one sex partner	1	2	3	4
4	Men feel ashamed of their wives and want young lovers to take around to their friends	1	2	3	4
5	If men do not have lovers their friends laugh at them	1	2	3	4
6	Women who are financially independent do not want to commit themselves to one relationship	1	2	3	4
7	The families of young people who work do not want them to get married because they are afraid to lose their income	1	2	3	4
8	Men often force women in subtle ways to have sex with them even if they do not want to	1	2	3	4

### Statistical analysis

STATA (version 14, StataCorp LP, College Station, TX) was used for the analysis. The main independent variable was beliefs on gender roles. To describe the enrolled sample, we used means (± standard deviations) or medians with associated interquartile ranges (IQR) for continuous variables depending on their distribution. We calculated frequencies and proportions for categorical variables with the Fisher exact test or chi-square test for comparisons. For the bivariate analysis we used the Fisher exact test for cell counts of less than 5. For larger cell counts per variable we used chi-square tests. p-values < 0.05 were considered statistically significant.

Factor analysis and Cronbach alpha measures were used to assess the reliability of the gender beliefs scale. For this scale, we summed responses across all eight items for each respondent and calculated a median score. Distribution of the summed scores were normal, suggesting that most of the beliefs on gender roles scores were within expected ranges. We used the median as an index of how progressive (promoting gender equality) or traditional (promoting male dominance) participants were with respect to gender roles. We generated a binary outcome score for each participant by summing scores from the eight-statements on gender beliefs as as progressive (scores of ≤ 16), or traditional (scores of > 16; scale range, 8–24) [34]. Scores for the progressive and traditional beliefs on gender roles were derived from the eight statements listed in Table 1. The binary score of yes or no as categorised using the cut-off point was used as the outcome variable while socio-demographic characteristics at baseline were used as the explanatory variables. A median score of 16 was used as the cut-off.

Multivariable logistic regression modelling was used to assess the main association of participant age and beliefs about gender roles (traditional versus progressive), adjusting for potential confounders. The outcome for the logistic regression model was traditional beliefs on gender roles. Potential confounders were defined a priori from review of the literature for demographics and behaviours that place men at high risk for HIV. Potential confounders included age, number of partners in the last 12 months, belief that last sexual partner was HIV positive and condom use at last sex act.

## Results

**Fig. 1:**
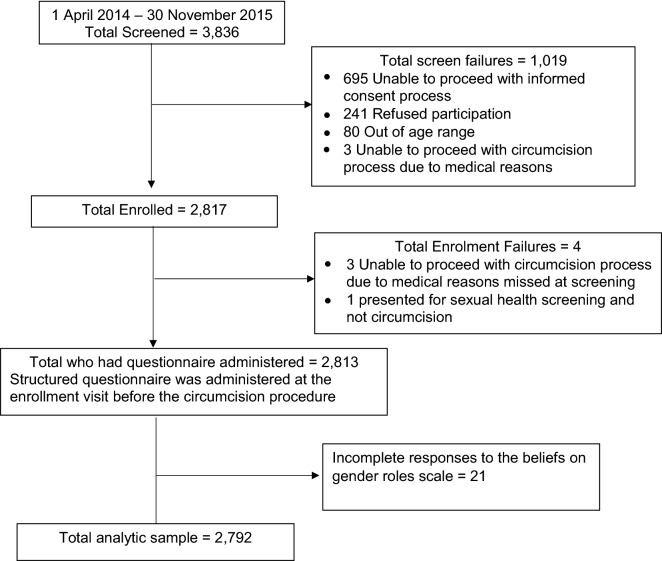
Enrolment diagram of men (aged 18–49 years) who accessed circumcision services between 2014 and 2015 in Ekurhuleni North, Gauteng Province, South Africa

Of the 3836 men screened during the *Imbizo*, study 1023 screened men were excluded (1019 after screening and 4 after enrolment). More than two-thirds of the screened participants (695 or 67.9%) could not proceed with the informed consent process as they were unable to communicate in any of the three study-supported languages and could not understand the consent form, 241 (23.6%) refused participation, 80 (7.8%) were out of the age range, 6 (0.6%) could not proceed with circumcision process due to medical reasons while one participant (0.1%) only attended the clinic for sexual health screening and not circumcision. The structured questionnaire was administered to 2813 (73.3%) eligible participants. For this analysis, 21 participant records were excluded due to incomplete information on the beliefs of gender roles scale. A total of 2792 participant records were used for this analysis (Fig. 1). The structured behavioural risk questionnaires took approximately 30 min to complete. Reliability for the scale on beliefs about gender roles was 0.6 (Cronbach alpha co-efficient). We observed that the 2792 analysed participants differed from the 21 excluded participants in terms of age. The group excluded were slightly older on average but no other significant difference was observed. Exclusion was mainly related to not being able to respond to all statements required on the beliefs of gender roles scale. Slightly older people were less likely to be able to complete all questions.

The overall median age was 26 years (interquartile range, 21–31 years). A total of 1330 (47.6%) participants had traditional beliefs about gender roles. Most participants were enrolled in phase one (1584; 56.7%), aged 25–34 years old (1173; 42.0%), and had steady employment (1259, 45.1%). A high proportion of participants reported a sexual relationship with at least one person over the last 12 months (1903; 68.2%); reported sexual debut at age ≤ 17 years (1579; 56.6%); did not believe that their last partner was HIV positive (2023; 72.4%); and whose last sex partner was ≥ 5 years older than the participant or the same age (1970; 70.6%) when the questionnaire was administered. Refer to Table 2.

**Table 2:** Sample characteristics and HIV risk behaviour at baseline and associations with beliefs about gender roles among men aged 18 to 49 years old (n = 2792) (2014–2015)

	Beliefs about gender roles		
	Progressive (n = 1462)	Traditional (n = 1330)	p-value
	n (%)	n (%)	
Phase of enrolment			
Phase 1 (1 Apr–30 Sep 2014)	800 (50.5)	784 (49.5)	0.02
Phase 2 (22 Jun–30 Nov 2015)	662 (54.8)	546 (45.2)	
Age			
18–24 years	600 (49.6)	609 (50.4)	0.02
25–39 years	757 (53.9)	647 (46.1)	
≥ 40 years	105 (58.7)	74 (41.3)	
Employment status			
Student	290 (53.8)	249 (46.2)	0.03
Unemployed^a^	380 (47.7)	416 (52.3)	
Some employment	104 (55.9)	82 (44.1)	
Steady employment	680 (54.0)	579 (45.9)	
Missing	8 (66.7)	4 (33.3)	
Alcohol use			
Never	522 (53.3)	457 (46.7)	0.31
Occasional (≤ 1 to 3 times a month)	676 (50.9)	652 (49.1)	
Frequent (Once a week to daily)	264 (54.4)	221 (45.6)	
Cannabis use			
No	1198 (52.5)	1085 (47.5)	0.19
Yes	264 (52.2)	242 (47.8)	
Missing	0 (0.0)	3 (100.0)	
Number of partners in the last 12 months			
0–1 sexual partner	1059 (55.7)	844 (44.4)	
> 1 sexual partner	402 (45.3)	485 (54.7)	< 0.01
Missing	1 (50.0)	1 (50.0)	
Age of sexual debut			
≤ 17 years	810 (51.3)	769 (48.7)	0.77
18–34 years	589 (53.6)	510 (46.4)	
Unknown age	7 (53.9)	6 (46.2)	
Never had sex^b^	53 (55.2)	43 (44.8)	
Missing	3 (60.0)	2 (40.0)	
Believe last partner was HIV positive			
No	1097 (54.2)	926 (45.8)	0.01
Yes	45 (45.9)	53 (54.1)	
Not sure^c^	263 (46.2)	306 (53.8)	
Not applicable^d^	55 (56.1)	43 (43.9)	
Missing	2 (50.0)	2 (50.0)	
Age of last sex partner			
> 5 years younger	387 (53.9)	331 (46.1)	0.41
Same age	981 (51.8)	914 (48.2)	
> 5 years older	37 (49.3)	38 (50.7)	
Not applicable^d^	55 (56.1)	43 (43.9)	
Missing	0 (0.0)	2 (100.0)	
Condom use at last sex			
No	727 (53.5)	632 (46.5)	0.06
Yes	680 (51.1)	650 (48.9)	
Missing	0 (0.0)	3 (100.0)	

Row percentages are shown

^a^Includes those that reported receiving social grants

^b^Total who never had sex is 98. There was 1 missing age and 1 data discrepancy for this variable

^c^Uncertain of partner’s HIV status

^d^These participants reported never having sex

Men enrolled in Phase 2 were more likely to have progressive beliefs about gender roles (p = 0.02). Men between 18 to 24 years old (p = 0.02), those with multiple partners (p < 0.01) and unsure of their last partner’s HIV status (p < 0.01) were more likely to have traditional beliefs on gender roles. Beliefs about gender roles were not significantly associated with participant’s age at sexual debut (p = 0.54), age of last sex partner (p = 0.49), or condom use at last sex act (p = 0.35). Although the phase of enrollment seemed relevant in the univariate model, we removed this variable from the multivariable model, as the phase of enrolment was closely correlated with participant age. Condom use at last sex act was not significant (p > 0.05) in the univariate model and was also removed from the multivariable model.

The final multivariable model (Table 3) showed that younger men (18–24 years old) were more likely to have traditional beliefs on gender roles (adjusted odds ratio [aOR], 1.5 [95% confidence interval (CI) 1.0–2.0]; p < 0.01) compared to men who were 40 years and above. Men with multiple partners (aOR, 1.5 (CI) 1.3–1.8]; p < 0.01) were more likely to have traditional beliefs on gender roles compared to men who reported none or only one partner. Those unsure of their last partner’s HIV status (aOR, 1.4 [95% CI 1.1–1.7]; p < 0.01) were more likely to have traditional beliefs on gender roles compared to those who who knew that their last sex partner was not HIV positive. While the aOR for men who knew their last partner was HIV positive was similar, there were few men in this strata and this did not achieve statistical significance.

**Table 3:** Logistic regression models of association between traditional beliefs about gender roles and HIV risk behaviour among adult men 18 to 49 years (n = 2783)

	Unadjusted odd ratio (95% CI)	p-value	Adjusted odds ratio (95% CI)	p-value
Phase of enrolment				
Phase 1 (1 April–30 September 2014)	1			
Phase 2 (22 June–30 November 2015)	0.8 (0.7–0.9)	0.02		
Age				
18–24 years	1.4 (1.0–1.9)	0.02	1.5 (1.0–2.0)	0.01
25–39 years	1.2 (0.9–1.7)	0.23	1.2 (0.9–1.6)	
≥ 40 years	1		1	
Number of partners in the last 12 months				
0–1 sexual partner	1		1	< 0.01
> 1 sexual partner	1.5 (1.3–1.8)	< 0.01	1.5 (1.3–1.8)	
Age of sexual debut				
≤ 17 years	1			
18–34 years	0.9 (0.8–1.1)	0.24		
Unknown age	0.9 (0.3–2.6)	0.85		
Never had sex^a^	0.9 (0.6–1.3)	0.45		
Believe last partner was HIV positive				
No	1		1	< 0.01
Yes	1.4 (0.9–2.1)	0.11	1.4 (0.9–2.1)	
Not sure^b^	1.4 (1.1–1.7)	< 0.01	1.4 (1.1–1.7)	
Not applicable^a^	0.9 (0.6–1.4)	0.71	0.9 (0.6–1.4)	
Age of last sex partner				
> 5 years younger	1			
Same age	1.1 (0.9–1.3)	0.33		
> 5 years older	1.2 (0.7–1.9)	0.45		
Not applicable^a^	0.9 (0.6–1.4)	0.67		
Condom use at last sex				
No	1			
Yes	1.1 (0.9–1.3)	0.22		
Not applicable^a^	0.9 (0.6–1.4)	0.61		

Models conducted for non-missing values

^a^Participants who never had sex

^b^Uncertain of partner’s HIV status

## Discussion

We found that young men with traditional beliefs about gender roles were more likely to report multiple sexual partners and were unsure of their last partner’s HIV status—factors that increase men’s HIV risk. Our findings suggest that MMC demand-creation campaigns and HIV prevention programmes could consider targeting men with traditional beliefs about gender roles who may be more likely to engage in high risk sexual behaviour.

Men aged 25 years and above are at high risk for HIV, yet many do not access HIV prevention services. While societal norms encourage men to have multiple partners [11, 26], this type of behaviour is associated with increased HIV risk [22, 25, 27]. Although our finding of multiple partners being a risk factor to HIV among men with traditional beliefs about gender roles concurs with other studies [15, 22–27], there was no significant association to not using condoms. This is possibly due to self-reporting bias that occurred with the questionnaire was administered. Biological and behavioural surveillance surveys conducted in Cape Town, South Africa, across 3 years (2006, 2008, and 2010), showed that the number of sexual partners reported by men has decreased [23]. Yet, multiple partners remains a driver for HIV infections. In our study, men with traditional beliefs about gender roles were also more likely to be unsure of the HIV status of their last sexual partner. Other studies have reported that when men control the sexual relationship, they often distrust their female sexual partners [7, 35] and deny their partner’s HIV status [21]. It is possible that female partners might not disclose their HIV status to male partners due to fear of discrimination, stigma, gossip, or abandonment [36, 37].

Men aged ≥ 40 years were more likely to have progressive beliefs about gender roles, and we saw a trend of men between the ages of 25–39 years falling between the younger men and the older men in terms of their beliefs. Age and gender role beliefs were also both correlated to the phase of study enrolment. We observed that men enrolled in the second phase of the study tended to be both more progressive and older (25 years and above); recruitment for this second phase put a greater emphasis on older men (25 years and above). The association between more progressive gender beliefs among those in phase two of enrolment may have been due to selection bias. From a societal perspective, it seemed that men who were especially concerned about their health to the point of pursuing MMC at an older age, were somehow also more progressive, forward-thinking, or otherwise different from the norm. This is consistent with diffusion of innovation theory, in which those most likely to try new things (“innovators and early adopters”) are usually a small, different portion of society [38], and from our findings mature men 40 years and above were more likely to have progressive beliefs compared to those who were younger. However, additional studies are needed to interpret this finding. Promoting gender equality behaviours (what we typically recorded as “progressive”), such as taking responsibility to protect partners from HIV [15] and maintaining personal respect [39], could improve health-seeking behaviours among men.

Our study had several limitations. We did not do a formal sample size calculation and our analysis was limited to the available data. Our sample was comprised of only men who participated in the study and were willing to be circumcised at a clinic and was not a random sample of all men from the general population, however we don’t have any reason to believe that these men are different from their demographic and risk behaviour to other men in the community. This study also was conducted in only one sub-district of South Africa, but we believe that it is representative of many other urban settings in South Africa. The internal consistency of the beliefs about gender roles scale in our analysis was modest (Cronbach alpha co-efficient of 0.6) and lower than the household survey that was used to standardise this Likert scale for use in South Africa. One possible reason for the difference in reliability between the studies is that the representative household survey included men and women [34], while our study only included men. Our work is also a cross-sectional study, and with the inherent problems of temporality, we are unable to properly assess whether correlates of gender beliefs are actually causal predictors. Another limitation was possible social desirability bias when responding to questions on sexual behaviour and/or beliefs on gender roles, however many men reported high risk sexual behaviour. Despite these limitations, this study documents the importance of understanding the association of men’s beliefs about gender roles and high risk sexual behaviour.

Our findings suggest that men with traditional beliefs about gender roles, and perhaps with an emphasis on younger (18–24 years old) men, should be targetted for HIV prevention programmes even among a clinic-based sample. Interventions that account for beliefs about gender roles among men who access MMC could help engage men and their partners in HIV prevention programmes and could promote gender equality behaviours.

## Conclusions

Our findings suggest that traditional beliefs about gender roles among men could drive the HIV epidemic in South Africa. Past studies have shown that men may change their perceptions on gender roles after circumcision [5, 39]. Future HIV prevention programmes could consider using circumcision clinics to involve men who seek MMC in discussing and developing interventions that promote gender equality. Multi-level interventions should also involve the sexual partners of men who seek MMC and key traditional stakeholders in the community. Awareness could be created in the community by educating men on the long-term negative impact of traditional beliefs about gender roles and associated high risk sexual behaviours. Such interventions could help decrease HIV transmission in South Africa.

## Data Availability

The datasets used and/or analysed during the current study are available from the corresponding author on reasonable request.

## Abbreviations

HIV: Human immunodeficiency virus
MMC: Medical male circumcision
UNAIDS: The Joint United Nations Programme on HIV/AIDS
STIs: Sexually transmitted infections
OR: Odds ratio
CI: Confidence intervals
IQR: Interquartile ranges
